# Pilot proteomics study for identifying a pollution exposure signature in firefighters

**DOI:** 10.1038/s41370-026-00889-0

**Published:** 2026-04-13

**Authors:** Victoria Y. Ding, Di Lu, Yinyao Ji, Mary M. Johnson, Francois Haddad, Juyong B. Kim, Benjamin D. Horne, Jin-Ah Park, Kari C. Nadeau, Manisha Desai, Holden T. Maecker

**Affiliations:** 1 Quantitative Sciences Unit, Department of Medicine, Stanford University School of Medicine, Stanford, CA USA; 2 Department of Environmental Health, T.H. Chan School of Public Health, Harvard University, Boston, MA USA; 3 Division of Cardiovascular Medicine, Department of Medicine, Stanford University School of Medicine, Stanford, CA USA; 4 Human Immune Monitoring Center, Institute for Immunity, Transplantation, and Infection, Stanford University School of Medicine, Stanford, CA USA

**Keywords:** Air pollution, Wildfires, Workplace exposures, Exposomics

## Abstract

**Background:**

Recent studies showed that immune and cardiometabolic biomarker levels increased in individuals exposed to air pollution.

**Objective:**

We sought to identify a pollution exposure signature in a pilot cohort of 54 firefighters and controls in the Bay Area.

**Methods:**

We analyzed Olink-processed proteomics data under multiple approaches for feature selection, including the LASSO. We selected 67 biomarkers related to well-defined pathways for targeted analyses, whereas 1463 unique assays were considered for untargeted analyses.

**Results:**

Levels of angiopoietin 1 and hydroxyacylglutathione hydrolase were consistently higher in firefighters compared to controls. Moreover, matrix metalloproteinase-1, a protein that promotes tumorigenesis, was higher at the visit with greater acute exposure. Fifteen other key biomarkers were jointly identified under untargeted and targeted approaches. Regression analysis found that 10 biomarkers were numerically higher in firefighters with high exposure.

**Significance:**

These analyses demonstrate the potential utility of large-scale plasma proteomics in identifying a pollution exposure signature.

**Impact statement:**

Exposure to particulate matter leads to proteomic changes that are associated with adverse health outcomes. Applying regularization-based feature selection methods to high-dimensional data can identify a small subset of biomarkers for targeted monitoring efforts. Our findings improve understanding of the implications of air pollution for healthy adults and serve as a foundation to developing strategies for mitigating occupational exposure.

## Introduction

Wildfires have intensified in the past decade, resulting in lasting social and economic impact. Additionally, fine particulate matter and other toxicants from wildfire emissions have been associated with a range of respiratory and cardiovascular issues [1], while their immunologic effects remain underexplored. In our previous study, plasma samples collected during or immediately after the 2014 El Portal wildfire revealed that forest fire exposure in a 12- to 25-year-old cohort was associated with higher levels of interleukin-1β and C-reactive protein compared to non-smoke-exposed controls, suggesting a proinflammatory state after acute exposure [2]. The more recent 2020 wildfire season—the worst in California’s history and burning over 4.3 million acres [3]—furthered the opportunity to pilot novel technology and methods for investigating the impact of wildfire pollution on proteomic transducers of health.

Dimensionality reduction techniques, such as partial least squares discriminant analysis (PLS-DA), have traditionally been used in omics studies, although sparsity-promoting methods for biomarker discovery—notably the least absolute shrinkage and selection operator (LASSO)—have gained momentum. Using both classical and novel approaches, the present study seeks to identify a pollution exposure signature in firefighters, as well as to assess differences over time and by level of exposure. Biomarkers targeting well-identified pathways were gleaned from current literature, and we hypothesized that levels of inflammasome markers would be higher in firefighters compared to unexposed controls. This research adds to the expanding evidence on the health effects of wildfire smoke exposure and enhances the methodological resources available for investigating related issues.

## Subjects and methods

### Study population and data processing

Our study included 31 active-duty firefighters and 38 healthy civilian adults residing in the Bay Area, California. To minimize confounding effects from the healthy worker effect [4], we carefully screened control participants to exclude comorbidities and ensure comparability. Specifically, no participants had cardiovascular disease or cancer, were taking immunomodulatory drugs before their blood draw, had a smoking history, or were exposed to second-hand tobacco smoke.

Firefighters provided blood samples at two time points, from March to June 2021. Twenty-three non-smoke-exposed civilian participants, each assessed once during 2017–2021, were selected to maximize comparability to firefighters in case-control analyses. Fifteen additional smoke-exposed civilian participants, who were assessed twice over the course of one year (October 2019 to August 2020), were separately considered in longitudinal analyses. Demographics and clinical characteristics were collected on all participants. Level of exposure, as determined by the number of structural fires, was recorded for firefighters only. Samples were processed and stored according to published methods [5].

Plasma samples were processed using Olink Explore, a high-multiplex, high-throughput protein biomarker platform that measures the relative concentration of proteins in liquid biopsies using next-generation sequencing and operating under a semi-automated protocol. Up to 1536 assays are generated across four panels—Cardiometabolic, Inflammation, Neurology, and Oncology. The assay was performed at the Stanford Genomics Center, according to protocols supplied by the vendor (Olink, ThermoFisher, Upsalla, Sweden). Per Olink Explore’s built-in process, each sample measurement was normalized to an internal control and log_2_ transformed. The median of the plate controls was then subtracted, and resulting values were intensity-normalized (i.e., autoscaled) to achieve mean 0 and standard deviation (SD) 1 for all markers. The intensity-normalized data were used for feature selection, whereas the log_2_-transformed data were used for assessing inter-plate variability.

Samples from firefighters and their unexposed controls were run on one plate, and samples from exposed controls were run on a second plate. Ten samples were run on both plates to assess inter-plate variability.

Targeted biomarkers were selected from the Olink panel based on previous studies investigating biomarkers of pollution exposure and biomarkers associated with survival and major cardiovascular outcomes from large epidemiology studies (Supplementary Fig. 1).

### Statistical analysis

Participant characteristics were summarized by case-control status. In primary analysis, pollution exposure signatures in firefighters were identified under both targeted and untargeted approaches in comparison to unexposed controls. Under each approach, we used *t*-tests with false discovery rate (FDR) controlled at 15% to assess marker-by-marker differences between firefighters and unexposed controls, as well as three multivariable techniques—the LASSO [6], elastic net [7], and PLS-DA [8]. Notably, the LASSO has been shown to yield sparse solutions compared to classical dimensionality reduction techniques [9], thereby allowing us to identify features with the largest and most consistent signal for future study. The LASSO is particularly effective in high-dimensional settings and helps to prevent overfitting by imposing a penalty on the regression coefficients. In our analyses, the value of the penalty parameter, λ, that minimized the binomial deviance was determined using 10-fold cross-validation. For elastic net, joint cross-validation for λ and the mixing parameter, α, was implemented using the ‘caret’ [10] workflow, which invokes the ‘glmnet’ [11] R package.

PLS-DA is an extension of principal components analysis that additionally considers the correlation between the dependent and independent variables when transforming the original data, i.e., “projection to latent structures.” Its sparse variant, sPLS-DA, used in our analysis prioritizes selection of the most discriminative features for sample classification [12]. We used 3-fold cross-validation repeated 50 times to determine the optimal number of components to retain and applied the variable importance in projection (VIP) > 1 criterion. The number of variables to select on each component and the prediction distance to evaluate the classification and prediction performance of sPLS-DA were also tuned. These steps were implemented using the ‘mixOmics’ [13] R package.

In secondary analyses, we separately evaluated targeted marker stability in firefighters and smoke-exposed controls over two time points using paired *t*-tests, with unadjusted *p*-values meant to be descriptive. Among firefighters, we also explored differences in biomarkers by exposure level (high vs. low, dichotomized based on the number of self-reported fire incidents to which they responded 30 days prior to blood draw, with high exposure defined as 4 or more incidents). Comparisons between high- and low-exposure groups were performed in the mixed effects model framework to utilize both time points and account for confounding by age. For each marker, the model included fixed effects of exposure status (high vs. low) and visit (1 or 2), and within-subject correlation was incorporated via a random effect. Estimated exposure group differences with 95% confidence intervals were presented as a forest plot for all targeted markers.

For quality control, we considered all markers included in Olink Explore 1536, as well as our curated list of 67 target markers. Consistent with Olink’s assay validation approach, we estimated Spearman’s rank correlation coefficient (a nonparametric measure of monotonic association, with values closer to ±1 indicating greater strength of rank correlation) for the subset of samples processed on multiple plates, with strength of correlation interpreted according to conventional thresholds [14]. All analyses were performed in the R statistical computing environment, version 4.3 (R Core Team, 2023).

## Results

Baseline characteristics of firefighters and unexposed controls (median age 41; 56% Non-Hispanic White) are summarized in Table 1, with standardized mean differences small (i.e., <0.5) across sex, ethnicity, and allergy history. Smoke-exposed controls were younger (median age 30) and otherwise similar to unexposed controls. Median (Q1–Q3) of service was 10 (5–18) years for the 16 firefighters in the low-exposure group and 18 (9–23) years for the 15 firefighters in the high-exposure group.

**Table 1:** Characteristics of participants with samples processed using Olink Explore.

Demographic characteristic	Primary analysis		Smoke-exposed control^a^, *N* = 15
	Firefighter^a^, *N* = 31	Unexposed control, *N* = 23	
Age, median [Q1, Q3]	41 [37, 49]	40 [34, 53]	30 [23, 33]
Male, *n* (%)	26 (84)	15 (65)	8 (53)
Race/ethnicity, *n* (%)			
Non-Hispanic White	16 (52)	14 (61)	11 (73)
Asian	9 (29)	5 (22)	4 (27)
Hispanic/Latino	6 (19)	2 (8.7)	0 (0)
Other	0 (0)	1 (4.3)	0 (0)
Unknown	0 (0)	1 (4.3)	0 (0)
Any allergies, *n* (%)	7 (23)	9 (39)	6 (40)

^a^These participants provided samples at multiple timepoints.

When considering samples collected at the initial visit, 22 biomarkers were selected by the LASSO, compared to 95 by PLS-DA and 294 by elastic net. Follow-up samples yielded 26 biomarkers selected by the LASSO, compared to 93 by PLS-DA and 158 by elastic net. The LASSO remained as expected as or more parsimonious than the other methods in targeted selection (4 vs. 10 and 14 at the initial visit and 9 vs. 9 and 29 at the follow-up visit). The 18 biomarkers concurrently selected by all multivariable methods, by visit and approach, are summarized in Table 2. Cells corresponding to selected biomarkers are labeled by whether univariate differences in biomarker levels with respect to unexposed controls were higher or lower in firefighters, with significant FDR-corrected differences indicated by an asterisk.

**Table 2:** Olink Explore-processed markers jointly selected by all multivariable methods when comparing firefighters to non-smoke-exposed controls, by study visit.

Olink Explore markers, by panel	Initial visit		Follow-up visit	
	Targeted	Untargeted	Targeted	Untargeted
**Cardiometabolic**				
G protein-coupled receptor 37		Lower*		Lower*
Granzyme H^a^	Lower*	Lower*	Lower*	
Pleiotrophin				Lower*
Surfactant protein D^a^	Lower*	Lower*		
**Inflammation**				
Angiopoietin 1		Higher		Higher
Interleukin-16^a^			Lower*	
Matrix metalloproteinase-1				Higher
Nibrin^a^			Lower*	
Neutrophil cytosolic factor 2				Lower
PC4 and SFRS1 interacting protein 1		Lower*		
**Neurology**				
CD8 alpha		Lower*		
Cytotoxic and regulatory T cell molecule		Lower*		
Peroxiredoxin 1				Lower*
T-cell leukemia/lymphoma 1A				Lower*
**Oncology**				
Beta-defensin 4A and 4B				Lower*
Fibroblast growth factor 21		Lower*		
Glucagon		Lower*		
Hydroxyacylglutathione hydrolase		Higher		Higher

Cells of selected markers are labeled as “Higher” or “Lower” based on relative abundance in firefighters compared to unexposed controls. Significant differences under univariate comparisons with the false discovery rate controlled at 15% using the Benjamini–Hochberg procedure are denoted by an asterisk (*).

^a^Marker on targeted list, as visualized in Supplementary Fig. 1.

At both visits, angiopoietin 1 (ANGPT1) and hydroxyacylglutathione hydrolase (HAGH) were consistently higher in firefighters compared to controls. At the follow-up visit with greater exposure to structural fires on average, matrix metalloproteinase-1 (MMP1) was also higher in firefighters. All but one of the remaining biomarkers represented in Table 2 were significantly lower in firefighters in univariate, FDR-corrected comparisons. In a sensitivity analysis that only considered optimally-matched pairs (Supplementary Table 1) with the pairing included as an unpenalized term in LASSO analysis, feature selection results remained largely consistent, with ANGPT1 and HAGH still found to be elevated in firefighters at both timepoints (Supplementary Table 2).

In the targeted biomarker analysis under a mixed effects regression framework, nine biomarkers showed significantly lower levels in the high exposure group, including macrophage migration inhibitory factor (MIF), granzyme H, and myeloperoxidase (MPO). Additionally, 10 biomarkers exhibited elevated levels in highly exposed firefighters, though these differences did not reach statistical significance. Among these, insulin-like growth factor binding protein 1 (IGFBP1), N-terminal pro-brain natriuretic peptide (NT-proBNP), and surfactant protein D (SFTPD) showed the largest age-adjusted differences. All age-adjusted exposure differences are summarized in Supplementary Fig. 2.

Between study visits (generally from lower to higher exposure to air pollution), firefighters witnessed significant increases in two biomarkers (MMP7 and NT-proBNP) and significant decreases in 11, including MIF and MPO. Smoke-exposed controls witnessed significant increases in five biomarkers (nibrin [NBN]; granzymes A, B, and H; interleukin-16 [IL16]) and significant decreases in six, including MMP7 and C-C motif chemokine ligand 5 (CCL5).

For the inter-plate comparisons on the 10 samples processed on different plates, the median (interquartile range) of estimated Spearman’s rank correlation coefficients, overall and among target biomarkers, were 0.70 (0.45–0.89) and 0.66 (0.47–0.81), respectively. These summaries indicate that most associations were in the moderate to strong range.

## Discussion

Our pilot study following a major wildfire season demonstrated the feasibility of timely participant recruitment and plasma sample processing using targeted proteomics, as well as the application of novel statistical approaches to high-throughput data. Notably, the LASSO’s penalty-based selection algorithm allowed us to concentrate future validation efforts on the top 2% of nearly 1500 assays.

Three biomarkers from inflammation and oncology panels were found to be higher in firefighters compared to unexposed controls. Prior studies have noted the roles of ANGPT1 upregulation in wound-healing [15] and of HAGH in the detoxification of reactive 2-oxoaldehydes [16]. Upregulation of MMP1, which enhances the migration of tumor cells and adversely associated with tumor outcome, has been implicated in 11 cancer types [17]. Evidence also suggests that members of the MMP family are involved earlier in tumorigenesis, i.e., malignant transformation, angiogenesis, and tumor growth at both primary and metastatic sites [18].

Among targeted biomarkers identified in secondary analyses to be numerically higher in firefighters with high exposure, NT-proBNP is a known prognostic indicator in chronic or acute heart failure [19], while SFTPD regulates inflammation at pulmonary sites and is a causal risk factor of chronic obstructive pulmonary disease [20]. Several biomarkers found to have increased with pollution exposure among firefighters and smoke-exposed controls (MMP7 [21], NBN [22], granzymes [23], and IL16 [24] have known roles in inflammation across multiple organ systems.

We anticipated that markers of the inflammasome pathway would be elevated in firefighters, especially among those with high exposure. Reflective of our efforts to ensure comparability between cases and controls, leptin levels—known to be inversely associated with physical fitness—did not significantly differ between firefighters and unexposed controls, although the firefighter cohort showed slightly lower levels on average and included greater representation of men. Among firefighters, those with higher exposure had numerically lower leptin levels than their lower-exposure counterparts. This could be due to differences in fitness or adaptation to repeat exposure. These observations highlight the inherent challenges of cross-sectional studies involving firefighters (who generally have higher fitness levels and potentially better metabolic health than the general population) and suggest the most appropriate study design to be longitudinal. In addition, controlling for fasting state, time of day of sampling, and activity level in future studies would minimize confounding. Finally, accounting for personal protective equipment use (e.g., self-contained breathing apparatus), duration of exposure, and type of firefighting tactics utilized would more accurately characterize firefighters’ exposure level.

After a five-year data collection effort, the Occupational Requirements Survey from the Bureau of Labor Statistics recently identified the firefighting occupation as the most physically demanding among nearly 500 U.S. jobs [25]. Despite this high level of physical fitness, comparisons between firefighters and unexposed controls, as well as between high- and low-exposure groups, revealed biomarkers associated with adverse health effects. These findings suggest that exceptional physical fitness alone may not provide sufficient protection against the health impacts of air pollution.

In future studies, longer follow-up with additional sample collection time points during and outside of wildfire season could establish within-participant biomarker changes and inform the degree of recovery or chronic pathology between exposures to smoke. Additionally, subgroup analyses conducted on a larger and more diverse cohort will provide insights into proteomic variations within the exposed population, especially among subgroups more vulnerable to PM_2.5._

## Supplementary information

Supplementary information

## Data Availability

The datasets analyzed during the present study are available from the corresponding author upon reasonable request.
