# Supplementary material for: Pilot proteomics study for identifying a pollution exposure signature in firefighters
Source: J Expo Sci Environ Epidemiol. 2026 Apr 13;36(6):1144–8. doi: 10.1038/s41370-026-00889-0 (PMC13727676; doi:10.1038/s41370-026-00889-0)
Supplement: Supplementary file 1 [file 41370_2026_889_MOESM1_ESM.docx]

**Supplementary Material**

**Supplemental Figure 1.** Schematic diagram of target biomarkers curated from literature.

**Supplemental Table 1.** Characteristics of age- and sex-matched participants with samples processed using Olink Explore.

| **Characteristic** | **Firefighter**  **N = 23** | **Unexposed Control**  **N = 23** |
| --- | --- | --- |
| **Age**, median [Q1, Q3] | 41 [34, 50] | 40 [33, 54] |
| **Male**, n (%) | 20 (87) | 15 (65) |
| **Race/ethnicity**, n (%) |  |  |
| Non-Hispanic White | 9 (39) | 14 (61) |
| Asian | 9 (39) | 5 (22) |
| Hispanic/Latino | 5 (22) | 2 (8.7) |
| Other | 0 (0) | 1 (4.3) |
| Unknown | 0 (0) | 1 (4.3) |
| **Any allergies**, n (%) | 6 (26) | 9 (39) |

**Supplemental** **Table 2.** Markers selected by all multivariable methods when LASSO regression is performed on age- and sex-matched firefighters and controls.

| Olink Explore markers, by panel | **Initial visit** | | **Follow-up visit** | |
| --- | --- | --- | --- | --- |
|  | Targeted | Untargeted | Targeted | Untargeted |
| **Cardiometabolic** |  |  |  |  |
| G protein-coupled receptor 37 |  |  |  |  |
| Granzyme H‡ |  |  |  |  |
| Surfactant protein D‡ |  |  |  |  |
| **Inflammation** |  |  |  |  |
| Angiopoietin 1 |  |  |  |  |
| Interleukin-16‡ |  |  |  |  |
| Nibrin‡ |  |  |  |  |
| PC4 and SFRS1 interacting protein 1 |  |  |  |  |
| **Neurology** |  |  |  |  |
| Cytotoxic and regulatory T cell molecule |  |  |  |  |
| Peroxiredoxin 1 |  |  |  |  |
| T-cell leukemia/lymphoma 1A |  |  |  |  |
| **Oncology** |  |  |  |  |
| Beta-defensin 4A and 4B |  |  |  |  |
| Fibroblast growth factor 21 |  |  |  |  |
| Glucagon |  |  |  |  |
| Hydroxyacylglutathione hydrolase |  |  |  |  |

‡Marker on targeted list, as visualized in **Supplemental Figure 1**.

Note: Cells of selected markers are shaded in red if relative abundance was higher in firefighters compared to matched controls and in blue if relative abundance was lower.

**Supplemental Figure 2.** Age-adjusted differences with 95% confidence intervals in biomarker levels between high and low exposure groups. Markers with significantly lower relative abundance in the high exposure group are highlighted in red.
